# Long-term consequences of arsenic poisoning during infancy due to contaminated milk powder

**DOI:** 10.1186/1476-069X-5-31

**Published:** 2006-10-31

**Authors:** Miwako Dakeishi, Katsuyuki Murata, Philippe Grandjean

**Affiliations:** 1 Department of Environmental Health Sciences, Akita University School of Medicine, 1-1-1 Hondo, Akita 010-8543 Japan; 2 Department of Environmental Medicine, Institute of Public Health, University of Southern Denmark, Winsløwparken 17, Odense C, DK-5000 Denmark; 3 Department of Environmental Health, Harvard School of Public Health, Landmark 3-111E, 401 Park Drive, Boston, MA 02215 USA

## Abstract

Arsenic toxicity is a global health problem affecting many millions of people. The main source of exposure is drinking water contaminated by natural geological sources. Current risk assessment is based on the recognized carcinogenicity of arsenic, but neurotoxic risks have been overlooked. In 1955, an outbreak of arsenic poisoning occurred among Japanese infants, with more than 100 deaths. The source was contaminated milk powder produced by the Morinaga company. Detailed accounts of the Morinaga dried milk poisoning were published in Japanese only, and an overview of this poisoning incident and its long-term consequences is therefore presented. From analyses available, the arsenic concentration in milk made from the Morinaga milk powder is calculated to be about 4–7 mg/L, corresponding to daily doses slightly above 500 μg/kg body weight. Lower exposures would result from using diluted milk. Clinical poisoning cases occurred after a few weeks of exposure, with a total dose of about 60 mg. This experience provides clear-cut evidence for hazard assessment of the developmental neurotoxicity. At the present time, more than 600 surviving victims, now in their 50s, have been reported to suffer from severe sequelae, such as mental retardation, neurological diseases, and other disabilities. Along with more recent epidemiological studies of children with environmental arsenic exposures, the data amply demonstrate the need to consider neurotoxicity as a key concern in risk assessment of inorganic arsenic exposure.

## Background

Arsenic toxicity is a global health problem affecting many millions of people. The major source of human exposure is contamination of drinking water from natural geological sources, but anthropogenic emissions from mining, smelting, or agricultural sources (pesticides or fertilizers) also contribute to local exposures [1]. Although current risk assessment is based on the recognized carcinogenicity of arsenic [2,3], other adverse effects, such as neurotoxicity, may also be relevant. For instance, peripheral neuropathy has been amply demonstrated in adults [4-6] and is thought to occur only at fairly high exposure levels that would already be deemed unacceptable from the viewpoint of preventing arsenic-induced cancer. In past incidents, 15 fatalities occurred among 500 patients exposed to arsenic-contaminated wine in France in 1888; 70 of 6,000 patients from arsenic-contaminated beer in England in 1900–1901; and 15 of 28 patients due to arsenic-contaminated cider in the US in 1924 [7]. These events involved adults only.

Developmental processes in the nervous system are vulnerable to disruption by such chemicals at doses that may not be toxic to mature systems [8-10], and consideration of developmental neurotoxicity would therefore seem to be appropriate. An extensive data base on developmental arsenic toxicity exists from an unfortunate poisoning incident in Japan in the mid-1950s. The so-called Morinaga dried milk poisoning has received only cursory coverage in the English-language scientific literature, but detailed accounts are available in Japanese. According to official records, more than one hundred infants died from arsenic poisoning [11], thus making the fatality rate of this food poisoning incident the most serious one ever to occur in Japan. In the absence of a detailed account in English, we therefore present an overview of the Morinaga dried milk poisoning based on reports published by Japanese researchers.

### Outbreak of Arsenic Poisoning in Japan

In the early summer 1955, physicians in the western part of Japan became worried about outbreaks of an unusual disease characterized by anorexia, skin pigmentation, diarrhea, vomiting, fever, and abdominal distention among infants, mostly less than 12 months of age [12,13]. In the beginning, before the etiology was recognized, most general practitioners attributed these illnesses to acute bronchitis or pneumonia and prescribed antibiotics, such as penicillin or streptomycin [7]. Pediatricians at a university hospital temporarily suspected Candida septicemia, as presence of *Candida albicans *in urine and skin was observed in some infants by microscopy. On August 20th, however, the autopsy on a 5-month-old infant did not provide any verification of either visceral Candidiasis or characteristic inflammatory changes in the liver.

As an apparent link, most of the patients were bottle-fed, often using the popular Morinaga dried milk brand. In this connection, Dr. Eiji Hamamoto, Professor of Pediatrics at Okayama University Hospital, thought that the clinical effects might have resulted from a metal or metalloid poisoning [7,14]. On August 23rd, Dr. Hamamoto further speculated that arsenic could be the culprit due to the specific clinical picture. He asked the department of Forensic Medicine of Okayama University to examine whether arsenic existed in the Morinaga dried milk, and this prediction was confirmed. On the following day, he presented the combined evidence to the public on behalf of the Director of the Health Department, Okayama Prefectural Government: (1) The patients showed fever, skin pigmentation, hepatomegaly, and anemia, which were all in accordance with clinical symptoms of arsenic poisoning; (2) arsenic was identified from the Morinaga dried milk that the patients had ingested; and, (3) although there was insufficient evidence for beneficial effects, administration of British anti-Lewisite (BAL) could be recommended as a tentative treatment against arsenic poisoning.

In the course of the day, the sale of this milk was banned in Japan. On the following day, arsenic was detected in the liver tissue and hair of a deceased infant. Due to BAL treatment, the patients' conditions improved, and the number of deaths immediately decreased [15]. In addition, after three mice were fed a diet with Morinaga dried milk for 7–9 days, histochemical examinations of the dead mice revealed detectable amounts of arsenic in the liver [7].

### Source of Arsenic Exposure

The arsenic poisoning was linked with MF-marked dried milk that was manufactured at the Tokushima plant (i.e., Morinaga F-plant). Morinaga records showed that the dried milk had been contaminated with arsenic from April 13th, 1955. The source was a disodium phosphate product added to cow's milk as a stabilizer to preserve constant acidity, but the particular industrial-grade disodium phosphate was of low purity and contained five to eight percent of arsenic (expressed as arsenous acid) [7,15]. When the disodium phosphate additive used at the plant was analyzed, it contained trisodium phosphate, sodium arsenate, and several other impurities. According to Nakagawa and Iibuchi [13], the additive was a byproduct of the process in which aluminum was produced from bauxite and consisted of approximately 45% crystal water, 14% P_2_O_5_, 28% Na_2_O, 2% V_2_O_5_, and 6% As_2_O_5_. At that time, the Tokushima plant manufactured approximately 200,000 one-pound cans of dried milk per month. The product was sold mainly in western Japan, and most of the victims accordingly resided in the same area [16]. Some Morinaga dried milk also appeared on the market of eastern Japan, including Tokyo, in August 1955, but most of the cans were fortunately collected before being sold.

The final arsenic concentration (expressed as arsenous acid) in MF dried milk was calculated to be between 0.001% and 0.007% (mean 0.003%) by the Hyogo Prefectural Institute of Hygiene and between 0.0015% and 0.0020% by the Tokyo Metropolitan Institute of Hygiene [12]. Although the arsenic content differed by lot number, MF dried milk can be assumed to contain about 21–35 μg arsenic per gram. Using the manufacturer's instructions printed on the label, final milk concentrations can be calculated to be 4.2–7.0 mg/L. For average milk consumptions, the daily arsenic intake would then be about 2.5 mg for a 1-month-old infant, 3.2 mg for a 2-month-old infant, and 4.6 mg for a 6-month-old infant [7]. On a body-weight basis, this dose corresponds to approximately 540 μg/kg body weight per day for 1-month-old infant, 590 μg/kg body weight per day for 2-month-old infant, and 610 μg/kg body weight per day for 6-month-old infant. If 50% milk or other diluted milk preparations were used, the arsenic exposures would be lower. Although the duration of exposure was generally not recorded, the appearance of cases during the summer would have allowed as little as a few weeks of exposure. More detailed information on total doses is available from descriptive studies of case series.

### Descriptive Studies of Acute Toxicity

**Figure 1:**
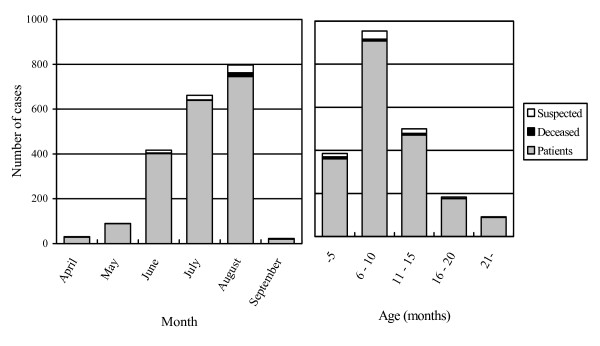
Distribution of 2,113 cases of dried milk poisoning in Okayama Prefecture in 1955.

In Okayama prefecture, poisoning cases occurred during the period of August 8th 1955 to April 30th 1956 and led to admission of 24 fatal cases, 2,005 surviving patients, and 84 suspected patients with some symptoms of arsenic poisoning but whose diagnosis was considered uncertain [7]. Of these 2,113 patients, infants aged 6–10 months predominated (N = 954), and males constituted 1,223 of all patients (57.9%) (Figure 1). The age distribution in the patients ranged from one month to 61 years. The first victim of MF dried milk seems to have appeared in April 1955, and the incidence reached the peak in July and August of the same year.

It was difficult to estimate the total amount of arsenic ingested in each case of MF dried milk poisoning, because arsenic content differed somewhat among lots of dried milk, the actual number of the dried milk cans purchased by each family was unknown, and dried milk was often consumed not only by infants but also by other household members. Based on data from patients whose diet was based only on MF dried milk, the critical dose causing clinical poisoning was estimated to be approximately five cans (i.e., 5 pounds of dried milk). From the manufacturer's instructions, a two-month-old infant would consume this amount in two-to-three weeks. The total dose can then be calculated to be about 60 mg.

Table 1 represents clinical symptoms and signs observed from 381 infant cases (320 outpatients and 61 inpatients) that were examined at the Okayama University Hospital [7]. All inpatients showed hepatomegaly but normal colloid reaction. Of the inpatients examined with electrocardiogram (ECG), almost half had abnormal ECG findings, such as ST elevation, flat T or prolonged QT interval. Table 2 shows results of clinical examination and chemical analyses from 59 inpatients [7]. Urinalysis showed erythrocyturia in several patients, while none had proteinuria. Unfortunately, neuromotor dysfunction or dysesthesia were impossible to assess in these pediatric patients.

**Table 1:** Clinical symptoms and signs observed in 381 infants with Morinaga dried milk poisoning*

	Number of patients with symptom	Frequency
Fever	358	94.0%
Diarrhea	269	70.6%
Vomiting	125	32.8%
Cough	134	35.2%
Eye discharge	141	37.0%
Skin pigmentation	349	91.6%
Skin rash	151	39.4%
Skin desquamation	182	47.8%
Edema	58	15.2%
Abdominal distension	105	27.6%

*Data from Okayama University Hospital [7]

**Table 2:** Results of clinical examination of 61 inpatients with Morinaga dried milk poisoning*

Parameter	Number abnormal among inpatients	Frequency
Hepatomegaly	61/61	100%
Electrocardiogram	26/57	45.6%
Red blood cell count		
<300 × 10^4^/μl	35/59	59.3%
<200 × 10^4^/μl	12/59	20.2%
White blood cell count		
<5000/μl	25/59	42.4%
<3000/μl	5/59	8.5%
Erythrocyturia	14/59	23.7%

*Data from Okayama University Hospital [7]

### Controversies About the Morinaga Dried Milk Poisoning

This poisoning incident happened during the early phase of Japan's rapid economic growth during the post-World War II period. At that time, most of the Japanese market for milk substitutes was covered by three major companies, including the Morinaga Milk Industry Company, and fierce competition demanded that production methods be optimized in regard to expenses. For this reason, food safety concerns seemed to be disregarded by the food manufacturing industry, judging from the fact that no quality control or assessment of raw materials was conducted by the company: Why did the Tokushima plant use a crude byproduct containing arsenic as an additive without due consideration? Also, if the company had used fresh milk for the production of dried milk, a disodium phosphate stabilizer would not have been required [15].

In the mid-1950s, the physicians concerned were not aware of any potential health risks associated with infant food. For this reason, only descriptive evidence was collected on clinical presentations until the time when arsenic was identified in the dried milk product. Likewise, even after the MF dried milk was banned, no research was carried out on arsenic levels in infant hair or urine. The clinical researchers involved in the Morinaga dried milk poisoning did not collect any data on exposure levels or tissue concentrations. As the result, the evidence of the Morinaga dried milk poisoning is severely limited in regard to documentation of exposures or dose levels, and no evidence is at hand to determine dose-response or dose-effect relations of arsenic exposure. A similar situation occurred later on in regard to Minamata disease (methylmercury poisoning) [17] and Kanemi rice oil poisoning (due to polychlorinated aromatic compounds, Yusho) [18].

On October 9th 1955, a child health committee, appointed by the Ministry of Health and Welfare, decided upon the formal criteria for diagnosis of poisoning caused by arsenic intake, as well as for identifying recovery [19]. The former criteria required skin pigmentation, hepatomegaly, and abnormal changes in blood cell values but was not based on drinking the arsenic-tainted Morinaga dried milk [19]. Some physicians pointed out that skin pigmentation, as compared to other symptoms, was not necessarily associated with the estimated intake of a toxic arsenic dose [15]. Thus, if a suspected case was to be registered without skin pigmentation, a high level of arsenic had to be proved in urine or hair of the case. The Ministry of Health and Welfare reported a total of 12,131 victims, including 130 fatalities by June 9th 1956; however, a number of unregistered patients were not included in the above figure [20], because most physicians did not request examination of urine or hair for arsenic, which was a tedious procedure at the time.

The ministerial committee judged that the disappearance of acute symptoms due to arsenic poisoning was regarded as a sign of complete cure. However, apparent resolution of acute toxicity, according to medical standards in Japan in those days, would be inadequate and erroneous as a criterion for complete recovery [15,19]. Thus, a coordinated follow-up study on child development should have been performed to determine the long-term consequences of slight brain edema, petecchial hemorrhage in the cerebellum, and myelin sheath degeneration of the optic nerves, which were observed on autopsy [7]. The news interest faded, and no further publications about the disaster appeared after the report by Hamamoto [7], thus reflecting the belief that the victims were thought to have recovered completely according to the above criteria [19].

Still, in March 1956, the Ministry of Health and Welfare sent the prefectural governments a notice requiring detailed medical checkups on patients with continued signs of Morinaga dried milk poisoning and on "cured" infants due to parental worries about prognosis and sequelae [11,15]. The patients were judged according to the presence or absence of acute effects of arsenic in regard to their possible need for further medical treatment and care, and most of them were therefore considered as cases without sequelae at the time of the checkup. Thus, no further follow-up was performed for many years.

In parallel, a third-party committee also favored the side of the enterprise (i.e., Morinaga Milk Industry Company) and did not recommend relief or support to the victims. Likewise, the first trial judgment in the Tokushima District Court applied the rule of reliance on reasonable conduct, and passed a sentence of not guilty on the offending company.

### Follow-up Studies

In 1969, Iibuchi and Nakagawa presented a study addressing the question "How are the infants who suffered from Morinaga dried milk poisoning 14 years ago?" at the meeting of Japanese Society of Public Health, and public attention was again paid to the patients. Thereafter, surveys of Morinaga milk poisoning patients began at several places of western Japan [15,19]. Since most of approximately twenty reports did not include any control groups or data on exposure levels, only two papers are reviewed here.

In Kyoto, epidemiological survey (415 subjects), clinical examination (292 subjects), and clinical psychological testing (261 subjects) were carried out during the period from December 1970 to July 1971 [15]. Poisoning victims in Kyoto had a much higher rate of physical and mental complaints than the control group; even average height in the victims was less than that of controls of the same age (Table 3). The prevalence of central nervous disorders, such as epilepsy, minimal brain damage or mental retardation, was increased in the victims. Also, the proportion of victims with intelligence quotient (IQ) less than 85 was higher than the anticipated from average values of the Japan Educational Ministry Statistics (Table 3). Based on these data, the authors concluded that the physical and mental defects of the victims were the result of consumption of arsenic-tainted Morinaga dried milk, and they emphasized that these children had received no therapy or support since then.

**Table 3:** Results of follow-up examination at adolescence of Morinaga dried milk poisoning victims in Kyoto [15]

Tests	Frequency abnormal (Number abnormal/total number)		Source of controls
		
	Victims	Controls	
Proteinuria			
Males	13.3% (25/174)	0.85%	16-year-old students in Kyoto
Females	11.7% (13/111)	1.20%	
Electroencephalogram	13.6% (39/287)	8.6%	1st grade high school students aged 16–18 years
Hearing disability	17.3% (50/289)	0.11%	16-year-old students in Kyoto
Refraction anomaly	47.9% (134/280)	41.77%	16-year-old students in Kyoto
Intelligence quotient <85	20.6% (52/252)	2.04%	Average value for Japan

Another comparative survey was made among all children born between January 1st, 1954 and December 31st, 1955 and brought up in a certain district of Hiroshima prefecture in 1971 [19]. The children were divided into three groups, namely those who had consumed the arsenic-tainted milk, those who were brought up on various brands of powdered milk from different companies, and those fed only maternal milk. At age 14 years, the victims had a lower IQ than their unexposed brothers and sisters. The victim group also had a higher rate of severe retardation (IQ below 50) than the controls who had not consumed the arsenic-tainted milk. Other, less remarkable findings among these children are shown in Table 4[19].

**Table 4:** Abnormal clinical findings from the follow-up study in Hiroshima [19]

Parameter	Number abnormal/total number (frequency)		
	
	Bottle-fed Morinaga milk	Bottle-fed other milk	Breast-fed
Dermatology	9/32 (28.1%)	5/26 (19.2%)	6/48 (12.5%)
Electrocardiogram	12/33 (36.4%)	8/25 (32.0%)	26/48 (54.2%)
Electroencephalogram	16/33 (48.5%)	8/26 (30.8%)	12/48 (25.0%)
X-ray of forearm and hand	12/29 (41.4%)	9/27 (33.3%)	24/48 (50.0%)
X-ray of foot	19/29 (65.5%)	23/27 (85.2%)	40/48 (83.3%)
Ophthalmology^a^			
Myopia	17/56 (30.4%)	20/52 (38.5%)	32/96 (33.3%)
Hyperopia	4/56 (7.1%)	4/52 (7.7%)	6/96 (6.3%)
Glasses-corrected vision <1.0	7/56 (12.5%)	2/52 (3.8%)	3/96 (3.1%)
Dentistry			
Periodontal disease	15/29 (51.7%)	15/26 (57.7%)	31/48 (64.6%)
DMF^b^	36.3%	35.4%	29.9%
Dentine abnormality^b^	14.2%	7.0%	11.1%

^a ^Denominator is the number of eyes

^b ^Proportion to the number examined teeth (DMF = decayed, missing, and filled teeth)

### Chronic Effects in Survivors of Morinaga Dried Milk Poisoning

The Hikari Association was established in April 1974, based on the agreement by a tripartite meeting (i.e., the victim group, Morinaga Milk Company, and Ministry of Health and Welfare), as a public-interest juridical foundation for relieving permanently the victims due to the Morinaga dried milk poisoning incident [21]. The Association reported that, by March 31st 2002, the total number of the victims was 13,420; approximately 6,000 of them have established contact with the Association. Of the 798 victims who have received welfare allowances from the Association in 2001, 337 suffered from developmental retardation; 129 suffered from various disabilities; 103 from mental disorders, and 33 from epilepsy.

## Discussion

The review of the unfortunate Morinaga dried milk poisoning incident leads to several observations. First, food safety considerations are imperative, in particular for large-scale production of infant foods. A simple review of the raw materials would have identified that the additive contained a toxic material that should not occur in a food product. Second, when infants sickened, the clinical facilities and the health authorities were not prepared to handle an epidemic, and countless numbers of cases probably occurred because the source of the toxicant exposure was only discovered with a substantial delay, despite the obvious connection with the milk substitute. Third, the clinical criteria established by the governmental authorities were rigid and misleading, and as a result, patients with chronic effects were ignored and were not provided with the treatment and support that they needed. Fourth, the failure to collect exposure information and the absence of registries and facilities for long-term follow-up of exposed population groups, such as the Morinaga victims, has made it impossible to determine the true extent of the adverse health effects in the victims. Finally, the victims of this unfortunate incident have not received the support and compensation that they deserved.

The Morinaga milk incident also has international aspects. Clearly, the unfortunate poisoning of thousands of infants was not heralded by governmental agencies as an illustrative case to support food safety measures. The failure to prevent the arsenic poisonings was considered embarrassing, and little effort was made to broadcast the lessons outside of Japan. Only brief reports in English have been published by Japanese scholars in international scientific journals [22]. However, these reports have been ignored by scientists abroad and by international agencies. As also noted elsewhere [10], the World Health Organization [3] and the US National Academy of Sciences [2] have recently published extensive risk assessment documents on arsenic, but developmental neurotoxicity is not offered a single sentence. Somehow, the world has therefore seemed to turn the blind eye to this very serious poisoning incident.

Arsenic is a well-documented cause of peripheral neurotoxicity in adults [4-6,22]. Recent cross-sectional studies of children at school age have reported cognitive deficits associated with increased drinking water contamination levels [23] and increased urinary arsenic concentrations [24]. Similar results were obtained in children with arsenic exposure from a smelter [25]. A possible joint adverse effect on IQ caused by arsenic and manganese exposures was suggested by hair contaminant concentrations in children living near a hazardous waste site [26]. These more recent data are externally consistent and fit with the high-exposure findings from Japan, although none of the above studies refer to the Japanese evidence.

The dose required to generate serious neurotoxic effects in the exposed Japanese infants was estimated to be of the order of magnitude of 60 mg, and the arsenic concentrations in the contaminated milk mixture (if not diluted) was estimated as 4.2 – 7.0 mg/L. This level may be compared to arsenic concentrations in contaminated drinking water in affected geographical areas, such as the Bengal, where the highest levels may approach or exceed 1 mg/L [3,24]. Although the Morinaga evidence does not allow a detailed dose-response relationship, the serious consequences in many victims suggest that the risk of developmental neurotoxicity must be taken into account when evaluating the potential adverse health effects of arsenic exposures.

## Conclusion

The Morinaga milk poisoning event provides clear-cut evidence for hazard assessment of developmental neurotoxicity. Clinical poisoning with severe neurobehavioral sequelae occurred when infants were exposed to total doses of about 60 mg from milk mixtures during a period of a few weeks. This evidence amply demonstrates the need to consider neurotoxicity as a key concern in regard to environmental arsenic exposure. Further, Morinaga evidence provides weighty support to several suggestive cross-sectional studies from various settings in regard to the subclinical neurotoxic effects of arsenic. The effects of arsenic on central nervous system development therefore need to be further explored and included in future risk assessments.

## Competing interests

The author(s) declare that they have no competing interests.

## Authors' contributions

The review was jointly conceived by KM and PG. MD was responsible for acquisition and collection of all references and for writing the first draft, under supervision by KM. All authors contributed to the revision of the manuscript, and all authors have given their final approval of the final version.
